# Return‐To‐Sport Assessments After Anterior Cruciate Ligament Injury: Which Jump‐Landing Test Is Sensitive to an ACL‐Injury History Under Fatigued or NonFatigued Conditions?

**DOI:** 10.1002/ejsc.12317

**Published:** 2025-05-10

**Authors:** Maité Calisti, Maurice Mohr, Felix Riechelmann, Inge Werner, Peter Federolf

**Affiliations:** ^1^ Department of Sport Science University of Innsbruck Innsbruck Austria; ^2^ Department of Orthopaedics and Traumatology Medical University of Innsbruck Innsbruck Austria

**Keywords:** classification, knee injury, principal component analysis, sensitivity analysis, whole‐body movement

## Abstract

Accurately identifying residual biomechanical deficits following an anterior cruciate ligament injury is critical for effective rehabilitation and safe return to sport. This study aimed to determine which of four jump‐landing tasks demonstrated the greatest sensitivity in distinguishing individuals with a history of ACL injury from healthy controls. Forty‐three participants formed the ACL (*n* = 21, 11 females) and the control group (*n* = 22, 12 females). Three‐dimensional motion data (Vicon, 250 Hz) were recorded during a single‐leg hop, unilateral countermovement jump, unilateral crossover hop, and medial‐rotation hop before and after a fatigue‐inducing intervention (single‐leg squats and step‐ups). Logistic regression models to classify participants were built using 13 lower‐body, trunk, and pelvis joint angles at 50 ms after initial ground contact, angular changes in these angles between 50 and 80 ms, and principal components derived from these variables. Classification rates and individual classification outcomes were assessed. The results revealed that no single jump‐landing task consistently outperformed others in detecting ACL injury history. Classification outcomes were influenced by fatigue state and analytical approaches. Fatigue was found to enhance classification rates. Combining joint angles with their temporal changes improved classification rates compared to using joint angles alone. However, applying principal component analysis as a preprocessing step did not consistently enhance model performance. Overall, the study demonstrated that jump‐landing tasks, combined with a variety of analytical approaches, can effectively detect ACL injury history. Fatigue enhanced classification outcomes, suggesting that it amplifies differences between post‐injury and healthy movement characteristics.

**Summary**

- The best‐performing jump test varied based on fatigue status, input data, and processing method.
- Classification rates were higher when participants performed jumps in a fatigued state, confirming the hypothesis that fatigue alters joint mechanics and enhances the detection of ACL injury history.
- Including changes in joint angles (between 50 and 80 ms) alongside joint angles at 50 ms after ground contact led to better classification rates.
- Athough principal component analysis improved classification outcomes in some cases, it did not universally enhance results, with some cases showing no change or decreased rates.

## 1 Introduction

Despite the progress in rehabilitation and return‐to‐sport (RTS) assessments following anterior cruciate ligament (ACL) injury, re‐injury rates remain high (Patel et al. 2021; Wiggins et al. 2016). Research indicates that up to 30% of individuals undergoing ACL reconstruction (ACLR) experience a second ACL injury, highlighting the need for more effective RTS assessment strategies (Paterno et al. 2014).

The RTS continuum, following an ACL injury involves a set of criteria used at the final stage of rehabilitation to determine if a patient is ready to safely resume physical activity or competition (Burgi et al. 2019). The most frequently used RTS criteria are time since the reconstruction, strength, and clinical examination, including general knee assessments post‐ACLR (Barber‐Westin and Noyes 2011; Burgi et al. 2019; Kaplan and Witvrouw 2019). The timing of RTS varies widely, ranging from 6 months to over a year (Lai et al. 2017), with an average reported duration of 7 months (Roi et al. 2005). Although hop tests are well‐documented in the ACL literature (King et al. 2018; Kotsifaki et al. 2022) and widely used throughout rehabilitation, the integration of movement quality analysis—such as joint angle assessments—is lacking in standard RTS decision‐making (Burgi et al. 2019). Burgi et al. (2019) reported that hop testing accounts for around 8%–10% of the total RTS criteria. When using hop testing, the performance differences—for example, in jump distance, jump height, and completion time—between the injured and uninjured limb are often used as a benchmark (Ardern et al. 2014; Kyritsis et al. 2016). The accepted level of asymmetry is a limb symmetry index (LSI) of at least 85% (Burgi et al. 2019). In the study of Burgi et al. (2019), over 75% of the reported studies analyzing strength and/or hop testing used an LSI cutoff to determine if an individual met the criteria for returning to sports. However, there is still no consensus on the thresholds and caution is necessary when interpreting the LSI (Bishop 2021), particularly if baseline measurements are unavailable (Wren et al. 2018), as LSIs frequently overestimate the knee function (Wellsandt et al. 2017). Furthermore, performance‐based LSIs do not directly quantify the quality of movement or the stability of the knee during a hop or jump‐landing task (Kotsifaki et al. 2020). Performance metrics like hop distance can also be easily manipulated resulting in an earlier clearance for RTS. Wren et al. (2018) showed that symmetric patients generally exhibited shorter hop distance compared to controls on both sides, suggesting that symmetry might be achieved, by reducing performance on the nonoperative side. Additionally, the uninjured contralateral limb may also display kinematic, stability, and performance impairments as a result of the contralateral injury (Calisti et al. 2023; Culvenor et al. 2016; Gokeler et al. 2017). This highlights the limitations of relying on the LSI and/or on performance criteria only, as it may overestimate progress in the ACLR limb (Patterson et al. 2020).

It is well‐documented that 9 months after ACLR, individuals still exhibit greater asymmetry in biomechanical variables compared to healthy controls (King et al. 2019). A study from Webster et al. (2021) showed that 3 years after ACLR, asymmetrical landing biomechanics persisted in a single‐limb landing assessment. However, caution is warranted when interpreting asymmetries in individuals with ACL injuries as asymmetries are also present in healthy individuals and asymmetry is a highly variable and often inconsistent metric, even in uninjured populations (Davey et al. 2021).

In contrast, Paterno et al. (2010) showed that changes in biomechanics at the hip and knee during a drop landing task were able to predict a second ACL injury, emphasizing the need to improve and test for movement quality in addition to jump performance during rehabilitation. One way to analyze joint angles is by assessing single‐joint alterations (Smeets et al. 2020). This method is frequently used, feasible for analyzing kinematics, and easily understandable for practitioners. However, evaluating just an isolated joint angle does not capture changes in whole‐body movement characteristics or the overall quality of a movement (Smeets et al. 2020). Another approach, allowing for a simultaneous assessment of multiple variables could be principal component analysis (PCA), which enables the identification of correlations between joint angles and the identification of kinematic patterns (Daffertshofer et al. 2004; O'connor and Bottum 2009). PCA is often used in human movement studies, for example, to detect motion synergies (de Freitas et al. 2010; Rethwilm et al. 2019) or as a preprocessing step in machine learning approaches (Bisele et al. 2017; Leporace et al. 2012; Mohr et al. 2019).

One method to assess movement quality in hop testing is through test batteries, including jump‐landing tasks and the use of 3D motion capture systems. There is a plethora of jump‐landing tests and variables described in the literature. Considering that time for clinical assessments through physicians or therapists is limited and valuable, it would be helpful to know more about the sensitivity of different jump tests and about the testing conditions in which they are most sensitive. Although some sensitivity assessments of jump tests already exist (Gustavsson et al. 2006; Itoh et al. 1998), they mainly include the analysis of asymmetries between injured and uninjured limbs and do not take into account the comparison to a healthy control group, nor do they analyze biomechanical parameters (Kotsifaki et al. 2021).

Finally, RTS decisions are typically made under nonfatigued conditions. However, research has shown that fatigued participants often fail to meet the required level for returning to sport (Augustsson and ThomeKarlsson 2004; Leister et al. 2018). In a nonfatigued condition, participants achieved the RTS criteria of > 90% LSI in a single‐leg hop test, whereas 68% showed a lower LSI when fatigued. Although findings vary (Barber‐Westin and Noyes 2017), most research indicates that acute fatigue leads to decreased neuromuscular function and unfavorable changes in jump‐landing biomechanics (Buckthorpe 2019). Several studies showed increased peak proximal tibial anterior shear force and decreased knee flexion angels, decreased hip flexion angle, increased knee valgus, and increased knee internal rotation when participants were fatigued (Borotikar et al. 2008; Chappell et al. 2005; Santamaria and Webster 2010; Webster et al. 2012).

In summary, jump landing tests play an increasingly important role in return‐to‐sport assessments after ACL injuries; however, a comprehensive comparative assessment of several jumps to identify the most suitable test under fatigued and nonfatigued conditions is, to the best of the authors’ knowledge, not yet available.

The primary objective of this study was to identify which of the four commonly used jump‐landing tasks demonstrated greatest sensitivity in distinguishing individuals with a history of ACL injury from healthy controls. The study employed a comprehensive approach combining multiple task conditions and analytical methodologies:

- Participants were tested in nonfatigued and fatigued conditions;
- Predictor variables included joint angle data captured 50 ms after touchdown versus the joint angle data and their change over time within a 30‐ms window (50–80 ms post landing);
- Predictor variables consisted of joint angles or principal component scores calculated from the joint angle data.

We hypothesized:

- Higher classification rates under fatigued compared to nonfatigued conditions;
- That the additional information regarding changes in joint angles over time results in higher classification rates;
- That the combined information captured in principal components facilitates higher classification rates than the joint angle data alone.

As a secondary objective, the classification results for the individual participants were compared between logistic models. It was hypothesized that some individuals are generally wrongly classified, suggesting that their kinematic movement patterns overlapped with the characteristics of the other group.

## 2 Methods

### 2.1 Participants

The study involved a total of 43 volunteers recruited from the entire student body of the university. The participants were physically active and participated in sports on several days per week (Table 1). The ACL group comprised 21 participants with a history of ACL injury and 22 healthy individuals in the control group (Table 1). The inclusion criteria were (1) absence of any lower extremity injuries in the previous 6 months and (2) absence of any previous severe ankle injury. Additional inclusion criteria for the ACL group were (1) one or two ACL injuries to the ipsilateral leg and (2) clearance to return to Level I sports (i.e., sports that involve jumping, hard pivoting, and cutting) for at least 1 year before participation in the current study. Beforehand, the study had been approved by the responsible ethics board (Certificate 98/2022). All procedures of the study were conducted following the ethical principles set down in the Declaration of Helsinki and all participants provided written informed consent before testing.

**TABLE 1:** Participant characteristics for the ACL and control group.^a^

	ACL group (*n* = 21; females: 11)	Control group (*n* = 22; females: 12)	*p*‐value*
Age (years)	24.2 ± 3.5	26.5 ± 3.2	0.008*
Height (m)	1.8 ± 0.1	1.7 ± 0.1	0.568
Weight (kg)	69.6 ± 10.7	68.0 ± 14.8	0.689
BMI (kg/m^2^)	22.6 ± 1.7	22.4 ± 2.5	0.350
ACLR	19	n/a	
ACLD	2		
Leg dominance^b^	Right: 19	Right: 20	
	Left: 2	Left: 2	
Injury side	Dominant leg: 9	n/a	
	Non‐dominant leg: 12		
Time since injury (year)	3.9 ± 2.5	n/a	
Reinjury on ipsilateral side	0/21	n/a	
Graft type	BPTB: 4	n/a	
	ST: 11		
	QT: 4		
Meniscus affected	Yes: 12	n/a	
	No: 9		
Physical activity (days/week)	4.9 ± 1.3	4.6 ± 1.1	0.399
Physical activity (minutes/session)	89.3 ± 34.0	94.1 ± 31.5	0.580
IKDC (%)	93.2 ± 7.8	n/a	
ACL‐RSI scale (score)	67.0 ± 17.9	n/a	
Tampa scale of kinesiophobia (score)	21.3 ± 4.8	n/a	
Jump height (cm)	Pre‐fatigue: 11.7 ± 4.3	Pre‐fatigue: 12.3 ± 3.2	Main effect fatigue: 0.001
	Post‐fatigue: 8.9 ± 3.8	Post‐fatigue: 9.6 ± 3.1	

Abbreviations: ACLD, anterior cruciate ligament deficiency; ACLR, anterior cruciate ligament reconstruction; ACL‐RSI scale, anterior cruciate ligament return to sport after injury scale; BPTB, bone–patellar tendon–bone graft; IKDC, International Knee Documentation Committee; QT, quadricep tendon graft; ST, semitendinosus‐gracilis tendon graft.

^a^ Values are represented as mean ± standard deviation for continuous data and as frequencies for nominal data.

^b^ Leg dominance was defined as the preferred kicking leg.

^*^ *p* < 0.05.

### 2.2 Experimental Protocol

Participant characteristics (Table 1) were determined. Before a standardized warm‐up, participants were given standardized footwear (Adidas Handball Spezial). A five‐minute warm‐up on a bike ergometer at body‐weight power and 80 rpm was conducted, followed by three submaximal bilateral and unilateral countermovement jumps (CMJ).

As a reference for the fatigue protocol, the maximal vertical jump height in a unilateral CMJ was measured. For the ACL group, this involved the injured leg, whereas for the control group, the leg was randomly selected. Participants were instructed to stand on a force plate, place their hands on their hips, and maintain their swing leg in 90° knee flexion. It was not allowed to use the swing leg for propulsion.

**FIGURE 1:**
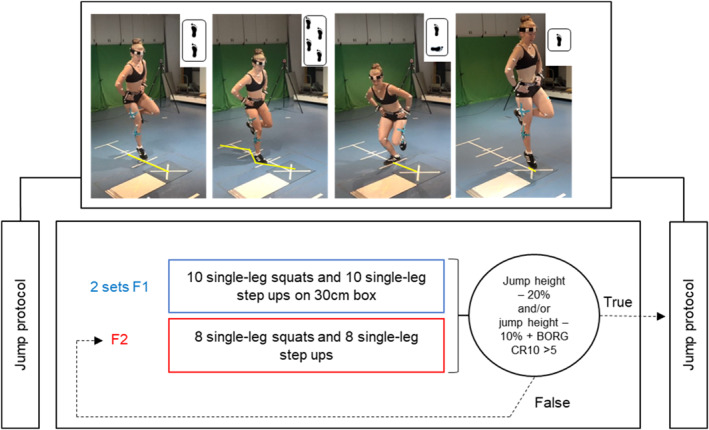
Top: the four jump‐landing tasks, from left to right: single‐leg hop, cross‐over hop, medial‐rotation hop, and countermovement jump. Bottom: the testing and fatiguing protocols: First, test jumps were performed in a randomized order, followed by fatiguing exercises F1. These were followed by a fatigue‐validation test (circle), which resulted either in advancing to post‐fatigue testing, or in further fatiguing exercises F2. F2 and the fatigue validation test were iterated until fatigue was confirmed. In post‐fatigue testing, two randomly selected jumps were performed, and then the fatigue exercises F2 were repeated again, followed by completing the remaining 2 jump tests.

For the jump protocol, participants performed four different jump‐landing tasks before and after a fatiguing protocol. The following tests were performed (Figure 1): single‐leg forward hop (SLH), single‐leg 90° medial‐rotation hop (MRH), single‐leg crossover hop (COH), and single‐leg CMJ. For the SLH, participants started at a standardized distance of 100% of their leg length (Webster et al. 2021) standing on one leg with their hands placed on their hips. Leg length was defined as the distance from the anterior superior iliac spine to the lateral malleolus. They were instructed to stand on the leg to be tested, hop in a forward direction, and land on the same leg onto the middle of the force plate (Noyes et al. 1991). For the MRH, participants were instructed to stand on the leg to be tested at a distance of 50% of their leg length, ensuring that the inner side of the foot is aligned perpendicular to the hopping direction. The hop was executed in the transversal plane, involving a 90° rotation in the medial direction during the swing phase. Upon landing, the foot was to be oriented in the forward direction (Dingenen et al. 2019). Hands had to be placed on the hips. For the COH, participants stood on the leg to be tested, with their hands placed on their hips, and performed three consecutive hops in the forward direction, crossing over a center strip on each hop and ending on the force plate (Noyes et al. 1991). For the CMJ, participants stood with the leg to be tested on the middle of the force plate, with their hands placed on their hips. The jump started with a countermovement to a self‐selected depth before accelerating in a vertical direction as fast and as high as possible (Bishop et al. 2020). They were additionally instructed to keep their swing leg in 90° knee flexion, and it was not allowed to use the swing leg as propulsion.

The jump‐landings were considered successful if the participants landed in the middle of the force plate, did not lose their balance, did not remove their hands from their hips, and held the final landing position in the lowest knee flexion position for at least 2 s. Data were collected for both legs, and three trials per leg were performed for all jumps, except for the COH jump where only one trial per leg was performed.

Following the completion of the jumps in the nonfatigued condition, the fatigue protocol was initiated (Figure 1). Participants performed two different sets (F1 and F2) of single‐leg squats and single‐leg step‐ups. For the single‐leg squats, participants lowered their bodies until their knees were at a 90° angle, lightly touching a 30‐cm box with their buttocks. The step‐ups were done onto a 30‐cm box. In set F1, participants completed two sets of 10 repetitions for each exercise. In F2, they performed eight repetitions for each exercise. After finishing the fatigue sets, the maximal jumping height was measured. Fatigue was defined by either (1) a 20% reduction in maximal jump height or (2) a 10% reduction in the maximal jump height along with a rating on the BORG CR10 scale higher than 5. If participants met either of these criteria, they immediately proceeded with two randomly selected jump‐landing tasks from the jump protocol. If none of the criteria were met, F2 was repeated until one of the criteria was fulfilled. To maintain the level of fatigue, F2 was repeated after performing two jump‐landings. The maximal jump height was calculated using the impulse–momentum theorem by a custom‐written LabVIEW program (Xu et al. 2023). The mean of three jumps was calculated to then define the 20% and 10% fatigue criteria.

### 2.3 Data Collection

Whole‐body kinematics and ground reaction force data were recorded using a 10‐camera motion tracking system (250 Hz; Vicon Motion Systems Ltd., Oxford, UK) and one force plate (1000 Hz; AMTI, MA USA). Additionally, a 2‐dimensional video camera (50 Hz, Vicon DV) recorded the testing procedure. In total, 59 retroreflective markers were placed on the participants (Vicon Plug‐in Gait full‐body model complemented with additional markers on the medial ankle and knee joint, greater trochanter, iliac crest, and marker clusters on the thigh and shank).

### 2.4 Data Analysis and Statistics

#### 2.4.1 Initial Data Processing

The trajectories of the retroreflective markers were initially reconstructed, and any gaps were filled through Vicon Nexus software v.2.14.0 (Vicon, Oxford, UK). Model scaling and inverse kinematics analysis were performed in OpenSim 4.3 using a modified version of the generic whole‐body musculoskeletal model of Catelli and colleagues (Catelli et al. 2019). It was subsequently modified to incorporate two additional degrees of freedom: knee abduction–adduction and knee internal–external rotation. Additionally, the ankle's pronation–supination degree of freedom was constrained. Further processing was done using a custom‐written Matlab script (The MathWorks Inc., Version R2021a, Natick, MA, USA).

Marker trajectories and force data were filtered using a third‐order, zero‐lag, low‐pass Butterworth filter with a cutoff frequency of 15 Hz. From OpenSim, 13 joint angles were extracted, including knee and hip flexion/extension angle, knee and hip abduction/adduction angle, knee and hip internal/external rotation, ankle flexion/extension angle, trunk flexion/extension, trunk bending, and trunk rotation, as well as pelvis tilt, pelvis list, and pelvis rotation. Since the fatigued/ACL leg was either the left or right leg for some participants, and the same applied to the control group, some joint angles needed to be mirrored.

These variables were calculated for the time point 50 ms after the initial contact (IC: > 20 N). This time point was selected since ACL injuries occur approximately within the first 50–100 ms during landing tasks (Bates et al. 2020; Krosshaug et al. 2007). Additionally, the angular change was calculated for all joint angles between 50 and 80 ms post‐IC.

#### 2.4.2 Classification Using Joint Angles (Pipeline “a” in Figure 2)

In order to obtain one value for each variable per person per trial, the mean over the three nonfatigued trials and over three fatigued trials was calculated for the SLH, CMJ, and MRH. Since for the COH only one trial per condition was performed, these single nonfatigued and fatigued trials were used for the analysis.

**FIGURE 2:**
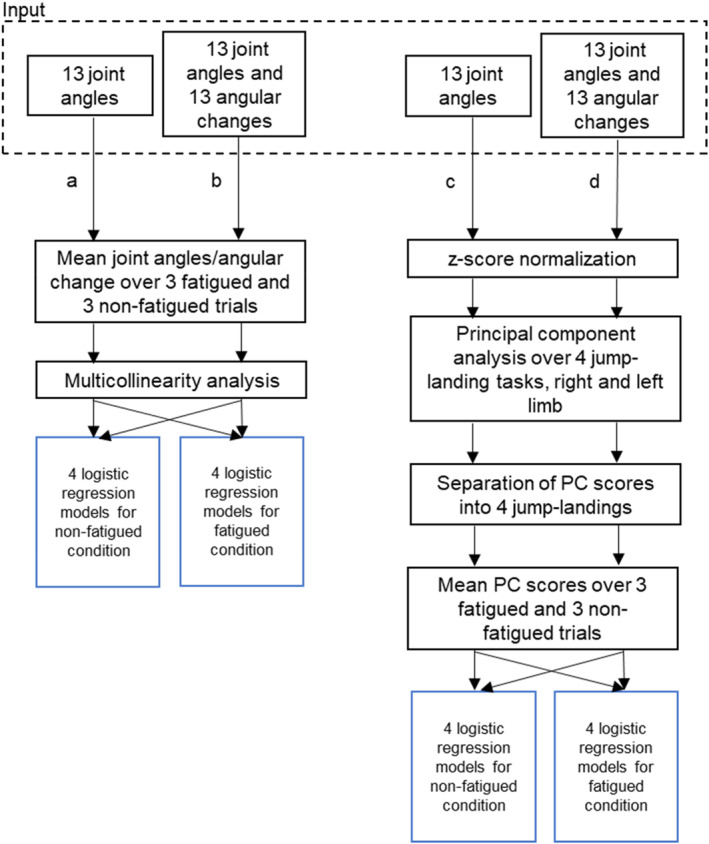
Data analysis flow diagram visualizing the four processing pipelines “a”–“d” that each resulted in eight logistic regression models (one for each: 4 analyzed jumps × 2 fatigue conditions) to determine how accurately participants could be classified into the ACL history and control group.

A correlation analysis was performed over the four jump‐landings between the 13 joint angles to detect and mitigate multicollinearity issues. This analysis led to the inclusion of 12 joint angles with pelvic tilt being excluded.

For the fatigued and nonfatigued conditions, and for each jump, a total of eight logistic regressions were performed where ACL history was treated as dependent and the 12 joint angles at 50 ms post‐IC served as predictor variables. A backward stepwise Wald method was employed. The final logistic model included only predictors (joint angles) needed for distinguishing the groups.

#### 2.4.3 Classification Using Joint Angles and Their Changes Over Time (Pipeline “b” in Figure 2)

In this analysis, in addition to the 13 joint angles at 50 ms post‐IC, the changes in angles within the 30‐ms time window were also considered. Again, mean angles over the 3 repetition trials were calculated and a multicollinearity analysis was conducted resulting in the exclusion of two potential predictors (pelvic tilt and angular change in pelvic tilt). Similarly, to the previous analysis, eight logistic regression models were determined using the backward stepwise Wald method.

#### 2.4.4 Classification Using Principal Components of Joint Angles (Pipeline “c” in Figure 2)

For the SLH, CMJ, and MRH, separate joint angle matrices M_1–3_ (where index 1 = SLH, 2 = CMJ, and 3 = MRH) were built consisting of 516 rows (one for each leg × 43 participants × 3 trials × 2 fatigue conditions) and 13 columns (13 joint angles). For the COH matrix, M_4_ consisted of 172 rows (only one trial per leg) and 13 columns.

Matrices M_1–4_ were normalized by subtracting the mean across observations and dividing the result by the standard deviation. For the PCA, M_1–4_ were concatenated to form a 1720 × 13 input matrix. The resultant PC scores were then separated for each jump and each condition (fatigued and nonfatigued). Then, mean PC scores were calculated for the three repetitions for each participant.

A collinearity analysis was not necessary since PC vectors are by definition orthogonal, resulting in PC scores that do not correlate strongly with each other. Analogous to the previous approaches, eight logistic models were then determined using the backward stepwise Wald method.

#### 2.4.5 Classification Using Principal Components of Angles and Their Changes (Pipeline “d”)

The input matrix for the second PCA‐based analysis was prepared analogous to the previous pipeline, but now contained the joint angles and their changes between 50 and 80 ms post IC, that is, they contained 26 instead of 13 rows. Again, eight logistic regression models—one for each jump and each fatigue condition—were derived from reducing the number of predictor variables through the backward stepwise Wald method.

### 2.5 Statistics

The binary logistic regression analyses were performed in SPSS Statistics 26 (IBM Corp., USA). The alpha level was set at *α* = 0.05. Chi‐squared tests were performed to assess the significance of the whole logistic model. Classification rates, sensitivity, and coefficient of determination (Nagelkerke *R* ^2^) were determined. We also report how many predictor variables were selected through the backward stepwise Wald algorithm. Reliability across the three trials was assessed using the two‐way mixed, absolute agreement intra‐class correlation coefficient (ICC, A, k) (McGraw and Wong 1996) and 95% confidence intervals (Supporting Information S1: Tables A5–A7). ICC can be interpreted based on Koo and Li (2016), where > 0.90 = excellent; 0.75–0.90 = good; 0.50–0.74 = moderate; and < 0.50 = poor. In addition, the mean and standard deviations of all variables across the three trials were calculated and are presented in Supporting Information S1: Tables A5–A7.

## 3 Results

The injured and control groups exhibited similar participant characteristics, except for age, which demonstrated a significant—yet clinically arguably negligible—difference (Table 1).

**FIGURE 3:**
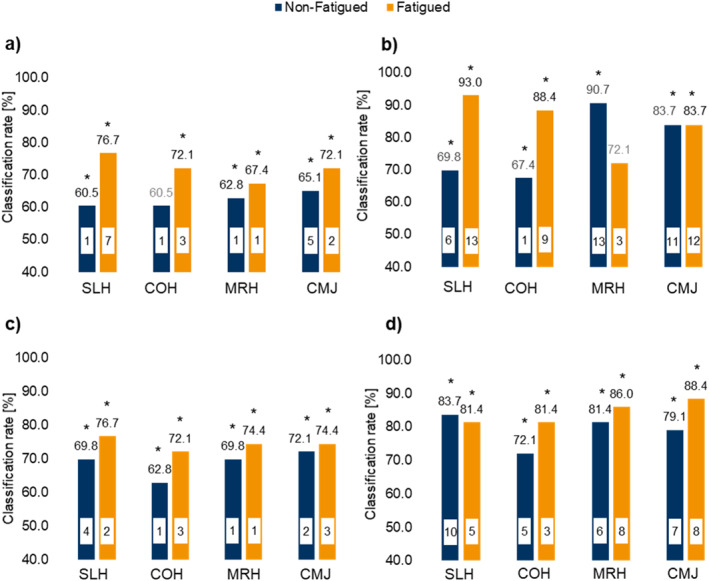
Correct classification rates (%) for the four jump‐landing tests: (a) based on joint angles, (b) based on joint angles and angular changes, (c) based on PC scores derived from joint angles, and (d) based on PC scores derived from joint angles and angular changes. Blue bars represent jumps in the nonfatigued condition, and orange bars represent jumps in the fatigued condition. CMJ, unilateral countermovement jump; COH, cross‐over hop; MRH, 90° medial‐rotational hop; SLH, single‐leg hop. White boxes within the bars indicate the number of predictors used in the logistic regression model. Asterisks (*) indicate significant logistic regression models.

An overview of all classification results is presented in Figure 3. It depicts four panels (a–d) which correspond to the processing pipelines (a–d) described in the methods and in the flow chart (Figure 2). Correct classification rates ranged from 60.5% to 93.0% obtained from logistic regression models that were statistically significant with only two exceptions (jump COH, nonfatigued in panel a; jump MRH, fatigued, in panel b).

Which jump performed best depended on the conditions (fatigued or nonfatigued), on the kinematic input data (joint angles or angles with their changes), on the data processing procedure, and apparently also on how many predictor variables were selected by the Wald algorithm. The highest classification rate across all processing pipelines was observed in the fatigued SLH, achieving a classification rate of 93.0% using joint angles and their changes over time (Figure 3, panel “b”). In the nonfatigued state, the MRH showed the highest classification rate of 90.7%, utilizing joint angles and their changes over time (Figure 3, panel “b”).

In the nonfatigued condition, classification rates ranged from 60.5% to 90.7% across all processing pipelines. For the fatigued condition, higher classification rates, ranging from 72.1% to 93.0%, were reached compared to the nonfatigued condition. In 13 out of 16 scenarios (combination of jump and analysis), higher classification rates were observed in the fatigued condition, except for the MRH and CMJ when using joint angles and their angular changes and for the SLH when using principal components of angles and their changes (Figure 3 and Supporting Information S1: Tables A1–A4).

Models incorporating joint angles and their angular changes achieved the highest classification rates, with the MRH reaching 90.7% in the nonfatigued condition and the SLH reaching 93.0% under fatigue compared to models using joint angles only or PC scores of angles and their changes. The models explained up to 83.2% of the variance (Nagelkerke *R* ^2^).

Performing a PCA on the angular and angular change data, Figure 3, panel “c” and “d”, respectively, yielded comparable or only slightly better classification rates than using the kinematic data directly as input for the logistic models. Here, the highest classification rate of 88.4% was observed in the fatigued CMJ.

Finally, generally higher classification rates were obtained when more predictor variables were selected by the algorithm. The number of selected predictors ranged from 1 to 7 out of 13 for the case where only angles were considered and from 1 to 13 out of 26 in the case that also changes in joint angles were included. All statistical model details, including the selected predictors and model accuracy quantifiers can be found in Supporting Information S1: Tables A1–A4.

### 3.1 Individual Classification Results

**FIGURE 4:**
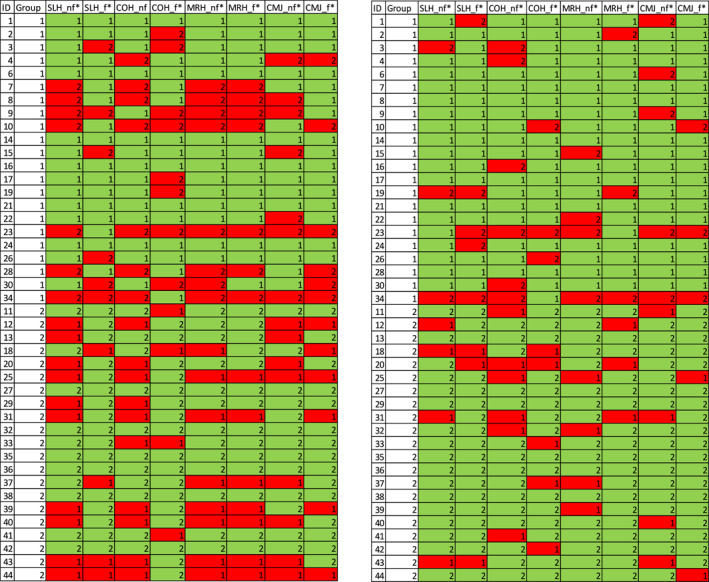
Individual correct classification results for the jumps SLH, CMJ, COH, and MRH for the nonfatigued (nf) and fatigued (f) conditions. A symbol “1” signifies the control group and “2” the ACL‐history group as predicted by the logistic regression model. The column “Group” is the actual group membership, and the colored columns indicate the classification outcome with green denoting a correct and red an incorrect classification. The left panel shows the results based on joint angles alone, which yielded the lowest classification rates, the right panel shows results for the PC‐score predictors calculated from joint angles and their changes within 30 ms.

When comparing individual classification results (Figure 4), it was found that different jumps could lead to different classification outcomes for the same individual, as did different fatigue states. Astonishingly, for each individual, at least one correct classification outcome was obtained in at least one jump or condition. There was no apparent systematic pattern. There were, however, individuals (e.g., IDs 23, 34, 20, and 31) who tended to be wrongly classified in a majority of cases. For others (e.g., IDs 7, 8, 9, and 10) classification tended to be wrong based on joint angles, but this issue was alleviated when the analysis was changed.

## 4 Discussion

This study investigated the sensitivity and classification rates using the lower limb and trunk kinematics observed in different jump‐landing tests under nonfatigued and fatigued conditions to distinguish between participants who had sustained an ACL injury and healthy controls. The main outcomes of the study are, first, that none of the four analyzed jumps stood out in terms of their ability to detect ACL injury history. The jump that performed best depended on the fatigue status, the selected input data, and the chosen processing approach. Second, the hypothesis that fatigued participants would enable higher classification rates was confirmed. Third, the hypothesis that considering changes in joint angles (between 50 and 80 ms) together with joint angles at 50 ms after ground contact would yield better classification rates than joint angles alone was also confirmed. Fourth, the hypothesis that performing a PCA as a preprocessing step would improve classification outcomes was not universally confirmed, as improvements were found in some cases, whereas in others, no changes or even decreases in classification rates were observed. Finally, in addition to the hypothesized influencing factors on classification rates, it was found that the number of predictor variables selected through the Wald procedure varied across cases, with a larger number of predictor variables generally leading to better classification results.

### 4.1 Suitability of the Different Jumps

Although none of the jumps stood out in all conditions, some advantages of individual jumps in specific conditions can be discussed. The SLH emerges as one of the most sensitive tests across all analyses, especially when fatigue and angular changes are considered. Its classification score reaches 93.0% in the fatigued condition when angular changes are included, highlighting its sensitivity to fatigue‐related movement adaptations.

When classifying without angular changes, the CMJ in the nonfatigued condition appears to achieve the highest classification rates. However, when angular changes are included in the analysis, the results become less consistent. Then, the MRH and the SLH seem to better distinguish the groups.

When it comes to physical performance tests for the knee, the single‐leg hop for distance is the most frequently studied (Grindem et al. 2011; Hegedus et al. 2015; Noyes et al. 1991). Although evidence regarding the validity of the hop test is mixed, with some studies suggesting it may not consistently reflect functional deficits, there is moderate support for its responsiveness to changes during rehabilitation (Hegedus et al. 2015). A sensitivity analysis by Gustavsson et al. (2006) demonstrated that the vertical jump, hop for distance, and side hop tests effectively distinguished between injured and uninjured limbs in patients 11 months post‐ACL injury and 6 months post‐reconstruction using the LSI. Still, comparing the ACL‐injured limb to a healthy, uninjured control may provide a more accurate assessment, as the LSI can sometimes overestimate the knee function by masking underlying deficits (Patterson et al. 2020). Prior research has questioned the sensitivity of the SLH when relying on hop distance as the primary outcome (Kotsifaki et al. 2020). In contrast, this study did not assess hop distance as a performance metric but instead standardized the jump distance to each participant's leg length to reduce variability and focus on movement quality. Kotsifaki et al. (2020) also reported that ACL‐injured individuals exhibit deficits in knee power absorption during landing. In the present study, the SLH showed the highest classification accuracy in distinguishing between the ACL‐injured and control groups, especially when fatigue and angular changes were included in the model. One possible explanation is that the fatiguing protocol used in this study specifically affected the quadriceps muscles and the neuromuscular control mechanisms required to stabilize the knee during SLH, particularly in the ACL group. This may have amplified compensatory landing patterns and contributed to the test's strong discriminative performance. These findings suggest that, when assessed through detailed kinematic analysis rather than outcome‐based metrics like hop distance, the SLH may still provide valuable insights into post‐ACL movement deficits and offer a clearer view of functional deficits than hop distance alone.

What all of the discussed studies share is their focus on performance variables such as hop distance or time. To gain a more comprehensive understanding of functional knee stability, incorporating kinematic variables would be highly beneficial. By analyzing joint angles, movement patterns, and other dynamic aspects of motion, we can obtain deeper insights into how the knee functions during physical tasks. This approach may offer a more detailed evaluation of knee stability beyond what simple performance metrics can provide.

### 4.2 Impact of Fatigue

Based on the correct classification rates across all analyses, we observe higher classification rates in 13 of 16 jump‐landings in the fatigued condition compared to the nonfatigued condition. In the fatigued condition, classification rates generally increase, suggesting more pronounced alterations in joint mechanics. The two exceptions to this finding, occurring in the MRH (Figure 3b) and in the SLH (Figure 3d), are most likely attributable to the substantially increased number of predictor variables in the nonfatigued cases and therefore have to be interpreted with caution. The fatiguing protocol used in this study was composed of repeated, body‐weight exercises (unilateral squats and step‐ups) designe to target the lower limb musculature. Fatigue in the quadriceps and hamstring muscles can disrupt neuromuscular control, which may contribute to altered movement patterns (Barber‐Westin and Noyes 2017).

Previous research indicates that fatigue can impact dynamic knee stability, impair motor control performance, and increase knee joint laxity (Benjaminse et al. 2008; P. T. Johnston et al. 2018; Rozzi et al. 1999). Regarding kinematic changes caused by fatigue, a study by Chappell et al. (2005) demonstrated significantly increased peak anterior shear forces on the proximal tibia, increased valgus moments, and reduced knee flexion angles during stop‐jump landings. Similarly, Mclean et al. (2007) found that under fatigued conditions, healthy participants exhibited significantly increased peak anterior shear forces on the proximal tibia, increased valgus moments, and decreased knee flexion angles during stop‐jump landings. In the current study, knee rotation emerged as a strong predictor in the models (Supporting Information S1: Tables A1 and A2), indicating that participants in this study continued to exhibit different kinematics compared to the healthy control group. For the classification based on principal component (PC) scores, the strongest predictors were PC3 and PC11. Of these, PC3 contained strong loadings for internal rotation and also for increased abduction angles. These patterns may reflect fatigue‐induced neuromuscular alterations, as fatigue might contribute to mediolateral instability due to altered motor control (R. B. Johnston et al. 1998), potentially compromising intermuscular coordination of the hip and thigh muscles.

### 4.3 Data Analysis Approach

The highest classification rates were observed in the models when the classification was performed using joint angles and their angular changes, with rates reaching between 83.7% and 93.0%. Further, when the classification was performed using PC scores derived from joint angles and their angular changes, rates between 81.4% and 88.4% were reached. Incorporating angular changes into the analysis improved the classification accuracy, especially under fatigue. By accounting for angular changes, the models incorporate more information about the jump landing movement, specifically the stiffness of the system during landing, making them more effective at distinguishing ACL‐injured individuals from healthy controls.

When classifying based on PC scores, the classification rates in both fatigued and nonfatigued conditions became more consistent across the jumps. This analysis found the highest classification rates when using the PC scores derived from joint angles and their angular changes, reaching rates in the nonfatigued condition between 72.1% and 83.7% and the fatigued condition between 81.4% and 88.4%. In contrast, decreased rates were observed when classifying using PC scores derived only from joint angles without considering how these angles change. Although PCA did not consistently improve classification rates compared to using joint angles directly, it still served as a valuable tool for identifying movement patterns. PCA is used in human movement analysis for identifying motion patterns (de Freitas et al. 2010; Rethwilm et al. 2019) and as a preprocessing step in machine learning approaches (Bisele et al. 2017; Leporace et al. 2012; Mohr et al. 2019). However, it is important to note that PCA is typically applied to waveform data rather than to discrete joint angles.

### 4.4 Influence of Number of Predictors

By using the Wald predictor selection method, we employed an objective, data‐driven approach to model building. This approach allowed us to systematically identify the most relevant predictors for distinguishing between participants with a history of ACL injury and healthy controls. We observed a trend where more predictors were selected for the SLH and CMJ, suggesting greater complexity in these statistical models. This may reflect higher complexity in the underlying differences in kinematic data that these tasks capture. Such complexity could arise from task‐specific movement patterns or compensatory strategies employed by participants with an ACL injury.

However, when a large number of predictors were selected, such as 13 in some models, we must acknowledge the risk of overfitting. Overfitting could reduce the generalizability of the model to new data. Furthermore, the trend of varying predictor numbers across models underscores the importance of task selection and methodological choices. These findings highlight the need for careful interpretation of the models with high predictor counts and emphasize the value of cross‐task comparisons.

### 4.5 Individual Classification Results

The variability in classification outcomes across different jumps and fatigue states suggests that no single jump or condition is universally superior for classification. Deficits after ACL injury may manifest uniquely in different individuals. However, the finding that every individual was correctly classified in at least one condition or jump suggests that combining multiple tasks and conditions might lead to more reliable assessments.

### 4.6 Strengths and Limitations of the Current Study

The current study offers several significant strengths compared to prior research on this topic. First, it is the most comprehensive to date in terms of the number of analyzed jump‐landing tasks, providing a broader evaluation of movement patterns relevant to ACL injury detection. We incorporated a variety of jump‐landing tasks, including a rotational jump, which is more challenging than forward or upward jumps, to provide a holistic view of task‐specific movements. Despite not requiring maximal forward jumps, we were still able to distinguish between the groups, emphasizing the importance of movement quality. Subtle differences in joint mechanics, control, and execution offer valuable insights, regardless of jump distance. Second, the study employed a carefully designed fatiguing protocol, ensuring robust and reliable testing of fatigue‐related effects on classification outcomes. Third, a variety of analytical approaches were systematically tested and compared, including joint angles, temporal changes in joint angles, and principal component analysis, offering novel insights into optimal strategies for distinguishing ACL participants from healthy controls.

Several limitations should be acknowledged. First, although the current study demonstrates that ACL injury history can often be successfully detected through jump‐landing assessments, it does not directly establish whether these jumps are suitable for return‐to‐sport assessments or return‐to‐sport decision‐making. Second, although the sample size in this study is comparable to or larger than that of similar studies, it remains limited, which constrains the generalizability of the findings and increases the risk of overfitting. Third, the variability in the number of predictors selected across models highlights the influence of task‐specific factors and analytical choices, which may limit the consistency and reproducibility of the findings in other contexts. Finally, although the choice of predictor variables and analytical approaches was comprehensive, it may not have captured all relevant biomechanical or neuromuscular factors. Incorporating additional predictors, such as full waveforms, muscle activity, or ground reaction forces, could further enhance the models' ability to identify ACL deficits.

## 5 Conclusions

This study demonstrated that jump‐landing tasks, combined with a variety of analytical approaches, can effectively detect ACL injury history in many cases. Fatigue was found to enhance classification outcomes, indicating that fatigue amplifies differences between post‐injury and healthy movement characteristics. This finding suggests that assessments in fatigued states should be incorporated into return‐to‐sport protocols to more reliably detect biomechanical deficits. Overall, the results also highlight the critical role of task selection, fatigue state, and analytical methods in shaping classification outcomes.

## Ethics Statement

Ethical approval was obtained from the Ethics Committee of the University of Innsbruck (Certificate 98/2022).

## Conflicts of Interest

The authors declare no conflicts of interest.

## Supporting information

Supporting Information S1
