# Supplementary material for: Return‐To‐Sport Assessments After Anterior Cruciate Ligament Injury: Which Jump‐Landing Test Is Sensitive to an ACL‐Injury History Under Fatigued or NonFatigued Conditions?
Source: Eur J Sport Sci. 2025 May 10;25(6):e12317. doi: 10.1002/ejsc.12317 (PMC12065478; doi:10.1002/ejsc.12317)
Supplement: Supplementary file 1 — Supporting Information S1 [file EJSC-25-e12317-s001.docx]

| Table A1. Classification using joint angle (pipeline “a”) in non-fatigued condition | | | | |
| --- | --- | --- | --- | --- |
| Non-fatigued | SLH (n = 43) | COH (n = 43) | MRH (n = 43) | CMJ (n = 43) |
| Predictors | Knee rotation | Knee rotation | Knee rotation | Knee rotation  Hip abduction  Hip rotation  Trunk bending  Pelvis rotation |
| Log-likelihood | 53.604 | 55.948 | 54.759 | 47.560 |
| Chi^2^ | 5.983 | 3.639 | 4.829 | 12.027 |
| Omnibus | .014 | .056 | .028 | .034 |
| Nagelkerke R^2^ | .17 | .108 | .142 | .325 |
| CR (%) | 60.5 | 60.5 | 62.8 | 65.1 |
| Sensitivity (%) | 52.4 | 52.4 | 61.9 | 61.9 |
| Specificity (%) | 68.2 | 68.2 | 63.6 | 68.2 |

| Table A1.1. Classification using joint angle (pipeline “a”) in fatigued condition | | | | |
| --- | --- | --- | --- | --- |
| Fatigued | SLH (n = 43) | COH (n = 43) | MRH (n = 43) | CMJ (n = 43) |
| Predictors | Knee rotation  Hip Flexion  Hip abduction  Hip rotation  Ankle flexion  Trunk extension  Pelvis rotation | Ankle flexion  Trunk extension  Pelvis list | Knee rotation | Knee rotation  Ankle flexion |
| Log-likelihood | 40.371 | 47.821 | 55.190 | 50.280 |
| Chi^2^ | 19.217 | 11.766 | 4.398 | 9.308 |
| Omnibus | .008 | .008 | .036 | .01 |
| Nagelkerke R^2^ | .48 | .31 | .13 | .26 |
| CR (%) | 76.7 | 72.1 | 67.4 | 72.1 |
| Sensitivity (%) | 81.0 | 81.0 | 66.7 | 71.4 |
| Specificity (%) | 72.7 | 63.6 | 68.2 | 72.7 |

| Table A2. Classification using joint angles and their changes over time (pipeline “b”) in non-fatigued condition | | | | |
| --- | --- | --- | --- | --- |
| Non-fatigued | SLH (n = 43) | COH (n = 43) | MRH (n = 43) | CMJ (n = 43) |
| Predictors | Ankle flexion  diff knee flexion | Knee rotation  Diff pelvis rotation | Knee flexion  Knee abduction  Knee rotation  Diff knee flexion  Diff hip flexion  Diff hip abduction  Diff trunk extension  Diff trunk bending  Diff trunk rotation  Diff pelvis rotation | Knee flexion  Knee abduction  Hip flexion  Ankle flexion  Trunk extension  Pelvis list  Diff knee flexion  Diff knee abduction  Diff hip flexion  Diff trunk rotation  Diff pelvis rotation |
| Log-likelihood | 46.412 | 51.151 | 28.318 | 25.548 |
| Chi^2^ | 13.175 | 8.437 | 31.269 | 35.039 |
| Omnibus | .040 | .015 | .002 | .015 |
| Nagelkerke R^2^ | .352 | .238 | .689 | .743 |
| CR (%) | 69.8 | 67.4 | 90.7 | 83.7 |
| Sensitivity (%) | 61.9 | 61.9 | 90.5 | 85.7 |
| Specificity (%) | 77.3 | 72.7 | 90.9 | 81.8 |

| Table A2.1. Classification using joint angles and their changes over time (pipeline “b”) in fatigued condition | | | | |
| --- | --- | --- | --- | --- |
| Fatigued | SLH (n = 43) | COH (n = 43) | MRH (n = 43) | CMJ (n = 43) |
| Predictors | Knee flexion | Hip abduction  Ankle flexion  Diff knee flexion  Diff knee rotation  Diff hip flexion  Diff ankle flexion  Diff pelvis list  Diff pelvis rotation | Ankle flexion  Diff ankle flexion  Diff pelvis list | Knee abduction  Knee rotation  Hip rotation  Ankle flexion  Trunk bending  Diff knee rotation  Diff hip rotation  Diff ankle flexion  Diff trunk extension  Diff trunk bending  Diff trunk rotation  Diff pelvis rotation |
| Log-likelihood | 17.524 | 22.419 | 51.983 | 27.600 |
| Chi^2^ | 42.063 | 37.168 | 7.604 | 31.987 |
| Omnibus | .001 | .001 | .055 | .001 |
| Nagelkerke R^2^ | .832 | .772 | .216 | .70 |
| CR (%) | 93.0 | 88.4 | 72.1 | 83.7 |
| Sensitivity (%) | 95.2 | 85.7 | 81.9 | 76.2 |
| Specificity (%) | 90.9 | 90.9 | 61.9 | 90.9 |

| Table A3. Classification using principal components of joint angles (pipeline “c”) in non-fatigued condition | | | | |
| --- | --- | --- | --- | --- |
| Non-fatigued | SLH (n = 43) | COH (n = 43) | MRH (n = 43) | CMJ (n = 43) |
| Predictors | PC3  PC5  PC10  PC12 | PC11 | PC11 | PC3  PC11 |
| Log-likelihood | 45.395 | 54.760 | 50.696 | 47.518 |
| Chi^2^ | 14.2 | 4.827 | 8.89 | 12.069 |
| Omnibus | .007 | .028 | .003 | .002 |
| Nagelkerke R^2^ | .38 | .142 | .249 | .33 |
| CR (%) | 69.8 | 62.8 | 69.8 | 72.1 |
| Sensitivity (%) | 71.4 | 66.7 | 71.4 | 71.4 |
| Specificity (%) | 68.2 | 59.1 | 68.2 | 72.7 |

| Table A3.1. Classification using principal components of joint angles (pipeline “c”) in fatigued condition | | | | |
| --- | --- | --- | --- | --- |
| Fatigued | SLH (n = 43) | COH (n = 43) | MRH (n = 43) | CMJ (n = 43) |
| Predictors | PC3  PC11 | PC3  PC5  PC10 | PC11 | PC3  PC10  PC11 |
| Log-likelihood | 45.588 | 47.135 | 51.941 | 43.113 |
| Chi^2^ | 13.9 | 12.452 | 7.647 | 16.475 |
| Omnibus | .001 | .006 | .006 | .001 |
| Nagelkerke R^2^ | .37 | .335 | .217 | .42 |
| CR (%) | 76.7 | 72.1 | 74.4 | 74.4 |
| Sensitivity (%) | 81.0 | 71.4 | 81.0 | 71.4 |
| Specificity (%) | 72.7 | 72.1 | 68.2 | 77.3 |

| Table A4. Classification using principal components of angles and their changes over time (pipeline “d)” in non-fatigued condition | | | | |
| --- | --- | --- | --- | --- |
| Non-fatigued | SLH (n = 43) | COH (n = 43) | MRH (n = 43) | CMJ (n = 43) |
| Predictors | PC1  PC2  PC3  PC6 PC9  PC10  PC21  PC23  PC24  PC25 | PC8  PC12  PC13  PC17  PC25 | PC1  PC9  PC16  PC19  PC20  PC21 | PC3  PC5  PC12  PC13  PC15  PC18  PC19 |
| Log-likelihood | 32.428 | 43.740 | 31.770 | 31.100 |
| Chi^2^ | 27.160 | 15.848 | 27.818 | 28.487 |
| Omnibus | .002 | .007 | <.001 | <.001 |
| Nagelkerke R^2^ | .624 | .411 | .635 | .646 |
| CR (%) | 83.7 | 72.1 | 81.4 | 79.1 |
| Sensitivity (%) | 81.0 | 71.4 | 81.0 | 77.3 |
| Specificity (%) | 86.4 | 72.7 | 81.8 | 81.0 |

COH: Classification with PC26 not possible, with PC25 possible

| Table A4.1. Classification using principal components of angles and their changes over time (pipeline “d)” in fatigued condition | | | | |
| --- | --- | --- | --- | --- |
| Non-fatigued | SLH (n = 43) | COH (n = 43) | MRH (n = 43) | CMJ (n = 43) |
| Predictors | PC12  PC16  PC18  PC19  PC20 | PC3  PC17  PC20 | PC3  PC8  PC9  PC17  PC18  PC24  PC25  PC26 | PC2  PC8  PC17  PC18  PC20  PC22  PC24  PC26 |
| Log-likelihood | 38.324 | 43.501 | 32.476 | 29.392 |
| Chi^2^ | 21.263 | 16.086 | 27.111 | 30.195 |
| Omnibus | .001 | .001 | .001 | <.001 |
| Nagelkerke R^2^ | .52 | .416 | .624 | .673 |
| CR (%) | 81.4 | 81.4 | 86.0 | 88.4 |
| Sensitivity (%) | 77.3 | 76.2 | 85.7 | 86.0 |
| Specificity (%) | 85.7 | 86.4 | 86.4 | 90.5 |

COH: Classification with PC26 not possible, with PC25 possible

| Table A5. Within session reliability, mean and standard deviation for the four jump-landing tasks for all joint angles and angular changes. | | | | | |
| --- | --- | --- | --- | --- | --- |
|  |  | Non-fatigued condition | | Fatigued condition | |
| Jump | Test Variable | Mean ± SD | ICC (95% CI) | Mean ± SD | ICC (95% CI) |
| SLH | *Joint angle 50ms:*  Knee flex/ext  Knee abd/add  Knee rot  Hip flex/ext  Hip abd/add  Hip rot  Ankle flex/ext  Trunk ext/flex  Trunk bending  Trunk rot  Pelvis list  Pelvis tilt  Pelvis rot  *Angular change 50-80ms:*  knee flex/ext  Knee abd/add  Knee rot  Hip flex/ext  Hip abd/add  Hip rot  Ankle flex/ext  Trunk ext/flex  Trunk bending  Trunk rot  Pelvis list  Pelvis tilt  Pelvis rot | 38.8 ± 7.0  0.3 ± 2.9  5.2 ± 7.7  46.1 ± 7.5  -9.1 ± 6.4  -1.5 ± 6.9  -2.8 ± 5.3  -18.1 ± 7.7  0.9 ± 3.9  5.7 ± 5.4  -11.2 ± 4.1  9.8 ± 5.4  -6.2 ± 5.4  13.0 ± 1.6  -1.2 ± 1.7  -0.1 ± 2.0  6.2 ± 2.0  4.1 ± 2.0  0.3 ± 2.8  5.2 ± 2.4  -1.3 ± 1.7  -3.1 ± 1.1  -2.5 ± 1.7  3.0 ± 1.0  0.0 ± 1.6  0.7 ± 1.4 | 0.75 (0.59, 0.86)  0.93 (0.88, 0.99)  0.97 (0.95, 0.98)  0.89 (0.81, 0.93)  0.86 (0.76, 0.92)  0.96 (0.93, 0.98)  0.88 (0.81, 0.93)  0.97 (0.94, 0.98)  0.78 (0.63, 0.88)  0.84 (0.73, 0.91)  0.83 (0.71, 0.90)  0.95 (0.91. 0.97)  0.85 (0.75, 0.92)  0.63 (0.38, 0.79)  0.83 (0.71, 0.90)  0.80 (0.67, 0.89)  0.85 (0.76, 0.92)  0.76 (0.60, 0.86)  0.85 (0.76, 0.92)  0.54 (0.24, 0.73)  0.75 (0.59, 0.86)  0.73 (0.55, 0.85)  0.84 (0.73, 0.91)  0.74 (0.56, 0.85)  0.89 (0.82, 0.94)  0.88 (0.79, 0.93) | 38.5 ± 7.0  0.2 ± 2.8  5.7 ± 8.3  45.9 ± 6.3  -9.0 ± 5.5  -1.3 ± 7.5  -2.5 ± 4.9  -18.2 ± 8.0  1.5 ± 4.2  6.6 ± 5.1  -11.4 ± 3.9  10.0 ± 5.5  -5.6 ± 5.4  13.3 ± 1.8  -1.2 ± 1.8  -0.4 ± 2.2  6.2 ± 2.2  4.1 ± 1.9  0.3 ± 2.9  5.2 ± 3.0  -1.6 ± 1.8  -3.3 ± 1.2  -2.4 ± 1.4  3.0 ± 1.1  -0.0 ± 1.7  0.7 ± 1.2 | 0.80 (0.68, 0.89) 0.92 (0.87, 0.95) 0.98 (0.97, 0.99)  0.90 (0.83, 0.94)  0.88 (0.80, 0.93)  0.97 (0.95, 0.98)  0.87 (0.79, 0.93)  0.97 (0.94, 0.98)  0.90 (0.84, 0.94) 0.84 (0.73, 0.91)  0.89 (0.82, 0.94)  0.94 (0.90, 0.96)  0.90 (0.83, 0.94)  0.74 (0.57, 0.85)  0.89 (0.81, 0.93)  0.87 (0.78, 0.92)  0.90 (0.83, 0.94)  0.80 (0.67, 0.89)  0.90 (0.83, 0.94)  0.65 (0.42, 0.80)  0.90 (0.83, 0,94)  0.79 (0.65, 0.88)  0.75 (0.59, 0.86)  0.78 (0.64, 0.88)  0.93 (0.89, 0,96)  0.81 (0.69, 0.89) |
| CMJ | *Joint angle 50ms:*  Knee flex/ext  Knee abd/add  Knee rot  Hip flex/ext  Hip abd/add  Hip rot  Ankle flex/ext  Trunk ext/flex  Trunk bending  Trunk rot  Pelvis list  Pelvis tilt  Pelvis rot  *Angular change 50-80ms:*  knee flex/ext  Knee abd/add  Knee rot  Hip flex/ext  Hip abd/add  Hip rot  Ankle flex/ext  Trunk ext/flex  Trunk bending  Trunk rot  Pelvis list  Pelvis tilt  Pelvis rot | 33.6 ± 5.3  1.4 ± 3.1  7.3 ± 8.1  33.9 ± 7.9  -12.1 ± 6.1  -4.1 ± 6.5  5.6 ± 8.9  -9.8 ± 8.3  7.9 ± 5.2  5.1 ± 5.0  -14.9 ± 5.0  17.2 ± 5.9  -0.1 ± 5.2  10.0 ± 3.1  -1.4 ± 1.3  0.4 ± 1.8  6.6 ± 2.9  5.1 ± 2.0  1.6 ± 2.4  9.6 ± 4.1  -1.3 ± 1.3  -3.2 ±1.3  -1.7 ± 1.5  2.8 ± 1.2  1.0 ± 1.3  1.4 ± 1.2 | 0.84 (0.73, 0.91)  0.92 (0.87, 0.96)  0.97 (0.95, 0.98)  0.90 (0.83, 0.94)  0.89 (0.82, 0.94)  0.91 (0.85, 0.95)  0.95 (0.91, 0.97)  0.96 (0.94, 0.98)  0.84 (0.74, 0.91)  0.72 (0.53, 0.84)  0.88 (0.79, 0.93)  0.89 (0.82, 0.94)  0.58 (0.29, 0.76)  0.88 (0.81, 0.93)  0.72 (0.54, 0.84)  0.65 (0.40, 0.80)  0.92 (0.86, 0.95)  0.82 (0.71, 0.90)  0.76 (0.60, 0.87)  0.91 (0.85, 0.95)  0.82 (0.70, 0.90)  0.83 (0.71, 0.90)  0.81 (0.68, 0.89)  0.80 (0.67, 0.90)  0.85 (0.74, 0.91)  0.82 (0.70, 0.90) | 32.5 ± 6.0  1.3 ± 2.5  6.7 ± 8.2  33.6 ± 7.6  -12.4 ± 6.0  -4.1 ± 6.6  5.7 ± 9.1  -9.8 ± 8.6  6.9 ± 4.9  4.8 ± 5.4  -14.7 ± 4.6  17.9 ± 5.8  -1.2 ± 5.1  10.0 ± 3.1  -1.4 ± 1.6  0.8 ± 2.2  6.8 ± 2.8  5.1 ± 1.9  1.4 ± 2.4  9.3 ± 4.5  -1.5 ± 1.4  -3.3 ± 1.4  -1.9 ± 1.5  2.9 ± 1.3  0.9 ± 1.2  1.5 ± 1.3 | 0.92 (0.86, 0.95)  0.93 (0.88, 0.96)  0.97 (0.95, 0.98)  0.92 (0.86, 0.95)  0.87 (0.78, 0.93)  0.93 (0.89, 0.96)  0.97 (0.94, 0.98)  0.96 (0.93, 0.98)  0.84 (0.74, 0.91)  0.80 (0.67, 0.89)  0.87 (0.78, 0.93)  0.89 (0.82, 0.94)  0.53 (0.22, 0.74)  0.88 (0.80, 0.93)  0.81 (0.68, 0.90)  0.78 (0.63, 0.87)  0.86 (0.81, 0.93)  0.85 (0.75, 0.91)  0.78 (0.64, 0.88)  0.92 (0.87, 0.95)  0.83 (0.71, 0.91)  0.88 (0.81, 0.93)  0.79 (0.65, 0.88)  0.89 (0.82, 0.94)  0.78 (0.63, 0.88)  0.82 (0.70, 0.90) |
| MRH | *Joint angle 50ms:*  Knee flex/ext  Knee abd/add  Knee rot  Hip flex/ext  Hip abd/add  Hip rot  Ankle flex/ext  Trunk ext/flex  Trunk bending  Trunk rot  Pelvis list  Pelvis tilt  Pelvis rot  *Angular change 50-80ms:*  knee flex/ext  Knee abd/add  Knee rot  Hip flex/ext  Hip abd/add  Hip rot  Ankle flex/ext  Trunk ext/flex  Trunk bending  Trunk rot  Pelvis list  Pelvis tilt  Pelvis rot | 38.3 ± 5.9  0.4 ± 3.1  7.6 ± 7.7  39.4 ± 7.9  -7.7 ± 5.8  1.7 ± 7.0  3.0 ± 5.4  -13.1 ± 8.2  1.6 ± 4.0  6.8 ± 6.2  -14.3 ± 4.3  8.7 ± 6.0  6.7 ± 6.6  10.8 ± 1.8  -1.1 ± 1.7  -1.6 ± 2.1  5.5 ±2.1  4.0 ± 1.9  0.6 ± 2.3  6.3 ± 2.2  -1.3 ± 1.7  -2.8 ± 1.4  -1.6 ± 1.4  3.0 ± 1.2  1.1 ± 1.5  -1.5 ± 1.2 | 0.90 (0.83, 0.94)  0.95 (0.91, 0.97)  0.97 (0.95, 0.98)  0.95 (0.92, 0.97)  0.90 (0.83, 0.94)  0.96 (0.94, 0.98)  0.94 (0.91, 0.97)  0.98 (0.97, 0.99)  0.85 (0.75, 0.92)  0.91 (0.85, 0.95)  0.89 (0.82, 0.94)  0.96 (0.94, 0.98)  0.83 (0.72, 0.90)  0.88 (0.79, 0,93)  0.83 (0.71, 0.90)  0.80 (0.70, 0.89)  0.88 (0.81, 0.93)  0.80 (0.66, 0.88)  0.85 (0.75, 0.91)  0.85 (0.74, 0.91)  0.92 (0.87, 0.96)  0.86 (0.77, 0.92)  0.76 (0.61, 0.87)  0.85 (0.76, 0.92)  0.91 (0.85, 0.95)  0.85 (0.74, 0.91) | 36.9 ± 5.7  0.7 ± 3.4  8.3 ± 8.4  38.7 ± 7.6  -8.0 ± 5.2  1.5 ± 7.1  2.4 ± 5.6  -13.5 ± 8.5  1.2 ± 4.0  8.2 ± 6.0  -14.3 ± 4.3  8.3 ± 5.7  7.6 ± 6.3  11.1 ± 1.9  -1.3 ± 1.6  -1.5 ± 1.9  5.9 ± 2.2  4.3 ± 1.8  0.4 ± 2.1  6.3 ± 2.7  -1.2 ± 1.4  -2.9 ± 1.4  -1.9 ± 1.4  3.2 ± 1.2  1.3 ± 1.5  -1.5 ± 1.2 | 0.88 (0.79, 0.93)  0.94 (0.89, 0.96)  0.97 (0.96, 0.99)  0.94 (0.90, 0.97)  0.88 (0.80, 0.93)  0.96 (0.93, 0.98)  0.93 (0.88, 0.96)  0.97 (0.95, 0.98)  0.77 (0.62, 0.87)  0.90 (0.83, 0.94)  0.89 (0.81, 0.94)  0.94 (0.90, 0.97)  0.84 (0.73, 0.91)  0.78 (0.63, 0.87)  0.81 (0.68, 0.89)  0.76 (0.60, 0.87)  0.91 (0.85, 0.95)  0.80 (0.66, 0.89)  0.75 (0.57, 0.86)  0.88 (0.80, 0.93)  0.87 (0.79, 0.93)  0.88 (0.80, 0.93)  0.79 (0.64, 0.88)  0.85 (0.75, 0.92)  0.90 (0.84, 0.95)  0.83 (0.72, 0.91) |
| COH | *Joint angle 50ms:*  Knee flex/ext  Knee abd/add  Knee rot  Hip flex/ext  Hip abd/add  Hip rot  Ankle flex/ext  Trunk ext/flex  Trunk bending  Trunk rot  Pelvis list  Pelvis tilt  Pelvis rot  *Angular change 50-80ms:*  knee flex/ext  Knee abd/add  Knee rot  Hip flex/ext  Hip abd/add  Hip rot  Ankle flex/ext  Trunk ext/flex  Trunk bending  Trunk rot  Pelvis list  Pelvis tilt  Pelvis rot | 39.9 ± 6.6  0.1 ± 3.3  5.1 ± 7.4  43.4 ± 9.2  -7.6 ± 6.5  -1.6 ± 7.1  0.3 ± 5.2  -17.1 ± 7.5  2.9 ± 4.7  4.8 ± 7.3  -12.1 ± 5.6  10.1 ± 5.8  -5.2 ± 6.7    11.7 ± 2.3  -0.9 ± 1.8  -0.0 ± 2.3  5.7 ± 1.8  2.9 ± 2.0  1.2 ± 2.5  5.6 ± 3.2  -1.2 ± 1.5  -2.5 ± 1.6  1.5 ± 1.4  2.1 ± 1.5  0.1 ± 1.3  0.3 ± 1.2 |  | 40.5 ± 6.6  0.0 ± 3.4  4.8 ±7.6  42.7 ± 7.6  -7.3 ± 5.5  -2.8 ± 7.4  0.5 ± 5.3  -17.5 ± 8.3  1.4 ± 4.3  4.9 ± 6.6  -10.9 ± 4.5  9.4 ± 5.9  -6.0 ± 6.6  11.9 ± 2.5  -0.9 ± 1.8  0.3 ± 2.3  5.7 ± 2.0  2.9 ± 2.0  0.6 ± 2.3  5.8 ± 2.7  -1.4 ± 1.6  -2.7 ± 1.5  -1.6 ± 1.6  2.1 ± 1.2  0.1 ± 1.4  0.4 ± 1.1 |  |

SLH = single leg hop; CMJ = countermovement jump; MRH = 90° medial rotational hop; COH = cross-over hop

| Table A6. Within session reliability, mean and standard deviation for the four jump-landing tasks for PC scores derived from joint angles. | | | | | |
| --- | --- | --- | --- | --- | --- |
|  |  | Non-fatigued condition | | Fatigued condition | |
| Jump | Test Variable | Mean ± SD | ICC (95% CI) | Mean ± SD | ICC (95% CI) |
| SLH | *PC scores of joint angles 50ms:*  PC1  PC2  PC3  PC4  PC5  PC6  PC7  PC8  PC9  PC10  PC11  PC12  PC13 | 0.3 ± 1.7  -0.2 ± 1.5  -0.0 ± 1.1  0.1 ± 1.1  -0.1 ± 1.0  -0.1 ± 0.8  0.0 ± 0.7  -0.1 ± 0.7  -0.1 ± 0.5  0.0 ± 0.5  0.0 ± 0.3  -0.0 ± 0.2  0.0 ± 0.1 | 0.82 (0.71, 0.90)  0.97 (0.95, 0.98)  0.93 (0.88, 0.96)  0.93 (0.88, 0.96)  0.81 (0.68, 0.89)  0.86 (0.76, 0.92)  0.93 (0.88, 0.96)  0.86 (0.77, 0.92)  0.81 (0.68, 0.89)  0.84 (0.73, 0.91)  0.79 (0.64, 0.88)  0.75 (0.58, 0.86)  0.82 (0.69, 0.90) | 0.2 ± 1.3  -0.2 ± 1.4  -0.1 ± 1.2  0.2 ± 1.2  0.0 ± 1.1  -0.0 ± 0.7  0.0 ± 0.7  0.1 ± 0.8  -0.0 ± 0.6  -0.1 ± 0.5  0.0 ± 0.3  -0.0 ± 0.2  -0.0 ± 0.1 | 0.85 (0.76, 0.92)  0.96 (0.94, 0.98)  0.95 (0.92, 0.97)  0.96 (0.94, 0.98)  0.90 (0.84, 0.94)  0.84 (0.73, 0.91)  0.88 (0.80, 0.93)  0.86 (0.77, 0.92)  0.85 (0.75, 0.91)  0.83 (0.71, 0.90)  0.84 (0.73, 0.91)  0.74 (0.57, 0.85)  0.87 (0.78, 0.92) |
| CMJ | *PC scores of joint angles 50ms:*  PC1  PC2  PC3  PC4  PC5  PC6  PC7  PC8  PC9  PC10  PC11  PC12  PC13 | 0.1 ± 1.8  -0.2 ± 1.3  -0.1 ± 1.1  0.4 ± 1.1  -0.0 ± 0.9  -0.0 ± 0.8  -0.0 ± 0.9  -0.0 ± 0.6  0.0 ± 0.6  -0.0 ± 0.4  -0.0 ± 0.3  0.0 ± 0.2  -0.0 ± 0.1 | 0.92 (0.86, 0.95)  0.92 (0.87, 0.96)  0.93 (0.87, 0.96)  0.88 (0.80, 0.93)  0.86 (0.80, 0.94)  0.68 (0.46, 0.82)  0.93 (0.88, 0.96)  0.76 (0.59, 0.86)  0.74 (0.57, 0.85)  0.65 (0.40, 0.80)  0.80 (0.67, 0.89)  0.68 (0.46, 0.82)  0.75 (0.58, 0.86) | 0.1 ± 1.7  -0.2 ± 1.3  -0.0 ± 1.1  0.2 ± 1.1  -0.1 ± 0.9  -0.0 ± 0.9  -0.0 ± 0.8  -0.1 ± 0.8  -0.2 ± 0.7  -0.0 ± 0.4  0.0 ± 0.3  0.0 ± 0.1  -0.0 ± 0.1 | 0.92 (0.87, 0.96)  0.93 (0.88, 0.96)  0.93 (0.88, 0.96)  0.87 (0.78, 0.92)  0.89 (0.82, 0.94)  0.72 (0.53, 0.84)  0.94 (0.90, 0.96)  0.85 (0.75, 0.91)  0.80 (0.67, 0.89)  0.79 (0.64, 0.88)  0.80 (0.67, 0.86)  0.64 (0.40, 0.79)  0.70 (0.50, 0.83) |
| MRH | *PC scores of joint angles 50ms:*  PC1  PC2  PC3  PC4  PC5  PC6  PC7  PC8  PC9  PC10  PC11  PC12  PC13 | 0.3 ± 1.6  -0.2 ± 1.5  -0.0 ± 1.0  0.2 ± 1.1  0.0 ± 1.0  -0.1 ± 0.8  0.0 ± 0.8  -0.1 ± 0.6  -0.0 ± 0.5  -0.0 ± 0.5  0.0 ± 0.3  -0.0 ± 0.2  -0.0 ± 0.2 | 0.92 (0.87, 0.96)  0.98 (0.96, 0.99)  0.94 (0.91, 0.97)  0.93 (0.89, 0.96)  0.90 (0.83, 0.94)  0.86 (0.75, 0.92)  0.95 (0.91, 0.97)  0.86 (0.76, 0.92)  0.85 (0.75, 0.92)  0.91 (0.84, 0.95)  0.82 (0.70, 0.90)  0.82 (0.69, 0.90)  0.82 (0.71, 0.90) | 0.1 ± 1.5  -0.3 ± 1.4  -0.0 ± 1.1  0.1 ± 1.3  -0.2 ± 1.0  0.1 ± 0.8  0.0 ± 0.8  0.1 ± 0.8  -0.0 ± 0.5  -0.0 ± 0.5  -0.0 ± 0.3  0.0 ± 0.2  -0.0 ± 0.2 | 0.88 (0.80, 0.93)  0.97 (0.94, 0.98)  0.96 (0.93, 0.98)  0.96 (0.92, 0.97)  0.90 (0.83, 0.94)  0.82 (0.69, 0.90)  0.93 (0.89, 0.96)  0.90 (0.83, 0.94)  0.77 (0.61, 0.87)  0.87 (0.80, 0.93)  0.80 (0.67, 0.89)  0.84 (0.73, 0.91)  0.85 (0.74, 0.91) |
| COH | *PC scores of joint angles 50ms:*  PC1  PC2  PC3  PC4  PC5  PC6  PC7  PC8  PC9  PC10  PC11  PC12  PC13 | 0.1 ± 1.8  -0.3 ± 1.4  0.0 ± 1.1  0.4 ± 1.6  0.0 ± 1.1  -0.1 ± 1.0  -0.1 ± 0.9  -0.0 ± 0.8  -0.0 ± 0.7  -0.1 ± 0.5  0.0 ± 0.4  -0.0 ± 0.2  -0.0 ± 0.2 |  | 0.3 ± 1.5  -0.3 ± 1.4  -0.1 ± 1.2  0.1 ± 1.3  -0.1 ± 1.1  -0.1 ± 0.9  0.0 ± 0.9  -0.0 ± 0.8  -0.0 ± 0.7  0.1 ± 0.5  -0.0 ± 0.4  -0.0 ± 0.2  -0.0 ± 0.2 |  |

SLH = single leg hop; CMJ = countermovement jump; MRH = 90° medial rotational hop; COH = cross-over hop

| Table A7. Within session reliability, mean and standard deviation for the four jump-landing tasks for PC scores derived from joint angles and angular changes. | | | | | |
| --- | --- | --- | --- | --- | --- |
|  |  | Non-fatigued condition | | Fatigued condition | |
| Jump | Test Variable | Mean ± SD | ICC (95% CI) | Mean ± SD | ICC (95% CI) |
| SLH | *PC scores of joint angles and angular changes:*  PC1  PC2  PC3  PC4  PC5  PC6  PC7  PC8  PC9  PC10  PC11  PC12  PC13  PC14  PC15  PC16  PC17  PC18  PC19  PC20  PC21  PC22  PC23  PC24  PC25  PC26 | -0.0 ± 1.8  0.3 ± 1.6  0.2 ± 1.4  0.0 ± 1.5  -0.2 ± 1.2  0.1 ± 1.2  -0.0 ± 1.0  0.0 ± 1.0  -0.1 ± 1.2  0.2 ± 0.7  -0.1 ± 0.7  -0.1 ± 0.9  -0.0 ± 0.7  -0.1 ± 0.6  -0.0 ± 0.5  -0.0 ± 0.5  -0.0 ± 0.4  -0.0 ± 0.3  -0.0 ± 0.3  -0.0 ± 0.3  -0.1 ± 0.3  0.0 ± 0.2  0.0 ± 0.2  0.0 ± 0.1  0.0 ± 0.1  -0.0 ± 0.1 | 0.77 (0.62, 0.87)  0.90 (0.84, 0.95)  0.85 (0.75, 0.91)  0.88 (0.81, 0.93)  0.88 (0.80, 0.93)  0.91 (0.85, 0.95)  0.88 (0.81, 0.93)  0.90 (0.83, 0.94)  0.87 (0.78, 0.92)  0.73 (0.54, 0.84)  0.77 (0.62, 0.87)  0.70 (0.50, 0.83)  0.78 (0.63, 0.87)  0.86 (0.76, 0.92)  0.84 (0.73, 0.91)  0.66 (0.43, 0.80)  0.86 (0.76, 0.92)  0.70 (0.49, 0.83)  0.75 (0.59, 0.86)  0.72 (0.54, 0.84)  0.79 (0.65, 0.88)  0.71 (0.51, 0.83)  0.67 (0.46, 0.81)  0.81 (0.68, 0.89)  0.76 (0.60, 0.86)  0.81 (0.69, 0.89) | 0.0 ± 1.7  0.3 ± 1.3  0.1 ± 1.6  -0.0 ± 1.4  -0.1 ± 1.2  0.0 ± 1.2  0.1 ± 1.0  0.1 ± 1.0  -0.1 ± 1.2  0.2 ± 0.9  -0.1 ± 0.8  -0.0 ± 0.9  0.0 ± 0.9  -0.0 ± 0.7  -0.0 ± 0.6  0.0 ± 0.6  0.1 ± 0.4  -0.1 ± 0.3  -0.1 ± 0.3  -0.1 ± 0.3  0.0 ± 0.3  -0.0 ± 0.2  0.0 ± 0.1  0.0 ± 0.1  0.0 ± 0.1  -0.0 ± 0.1 | 0.81 (0.69, 0.89)  0.89 (0.82, 0.94)  0.95 (0.92, 0.97)  0.86 (0.77, 0.92)  0.91 (0.84, 0.95)  0.95 (0.92, 0.97)  0.90 (0.84, 0.94)  0.93 (0.88, 0.96)  0.90 (0.84, 0.94)  0.82 (0.71, 0.90)  0.83 (0.72, 0.90)  0.70 (0.50, 0.83)  0.83 (0.71, 0.90)  0.85 (0.75, 0.91)  0.83 (0.72, 0.90)  0.77 (0.63, 0.87)  0.84 (0.74, 0.91)  0.62 (0.37, 0.78)  0.83 (0.71, 0.90)  0.62 (0.37, 0.78)  0.82 (0.70, 0.90)  0.81 (0.68, 0.90)  0.71 (0.52, 0.83)  0.80 (0.67, 0.89)  0.84 (0.73, 0.91)  0.87 (0.78, 0.92) |
| CMJ | *PC scores of Joint angle and angular changes:*  PC1  PC2  PC3  PC4  PC5  PC6  PC7  PC8  PC9  PC10  PC11  PC12  PC13  PC14  PC15  PC16  PC17  PC18  PC19  PC20  PC21  PC22  PC23  PC24  PC25  PC26 | -0.0 ± 1.7  0.2 ± 2.2  0.2 ± 1.4  -0.0 ± 1.2  -0.3 ± 1.2  -0.1 ± 1.3  0.0 ± 1.2  0.2 ± 0.9  -0.1 ± 0.8  0.2 ± 0.8  0.0 ± 0.7  0.0 ± 0.7  -0.0 ± 0.7  -0.0 ± 0.5  -0.0 ± 0.6  0.0 ± 0.5  -0.0 ± 0.4  -0.0 ± 0.3  -0.0 ± 0.3  0.0 ± 0.3  0.0 ± 0.2  -0.0 ± 0.2  -0.0 ± 0.1  -0.0 ± 0.2  -0.0 ± 0.1  -0.0 ±.0.1 | 0.82 (0.70, 0.90)  0.95 (0.91, 0.97)  0.83 (0.71, 0.90)  0.81 (0.69, 0.89)  0.84 (0.73, 0.91)  0.91 (0.85, 0.95)  0.90 (0.81, 0.94)  0.79 (0.64, 0.88)  0.85 (0.75, 0.92)  0.88 (0.80, 0.93)  0.67 (0.45, 0.81)  0.87 (0.78, 0.93)  0.81 (0.68, 0.89)  0.62 (0.37, 0.79)  0.76 (0.60, 0.87)  0.69 (0.48, 0.83)  0.75 (0.58, 0.86)  0.65 (0.41, 0.80)  0.66 (0.44, 0.81)  0.61 (0.35, 0.78)  0.69 (0.49, 0.82)  0.64 (0.39, 0.79)  0.59 (0.32, 0.77)  0.81 (0.67, 0.89)  0.67 (0.45, 0.81)  0.70 (0.49, 0.83) | 0.0 ± 1.6  0.2 ± 2.2  0.2 ± 1.4  0.0 ± 1.5  -0.1 ± 1.2  0.0 ± 1.3  0.1 ± 1.1  0.2 ± 0.9  -0.2 ± 0.8  0.1 ± 0.8  -0.1 ± 0.8  -0.1 ± 0.6  -0.0 ± 0.8  -0.2 ± 0.5  0.0 ± 0.6  -0.1 ± 0.5  0.0 ± 0.4  -0.1 ± 0.3  0.0 ± 0.3  0.0 ± 0.3  0.0 ± 0.3  0.0 ± 0.2  -0.0 ± 0.1  0.0 ± 0.2  0.0 ± 0.1  -0.0 ± 0.1 | 0.84 (0.73, 0.91)  0.94 (0.91, 0.97)  0.83 (0.72, 0.90)  0.89 (0.82, 0.94)  0.86 (0.79, 0.93)  0.91 (0.85, 0.95)  0.85 (0.73, 0.92)  0.73 (0.54, 0.85)  0.89 (0.82, 0.94)  0.82 (0.70, 0.90)  0.74 (0.57, 0.85)  0.87 (0.78, 0.92)  0.88 (0.79, 0.93)  0.67 (0.45, 0.81)  0.80 (0.63, 0.87)  0.76 (0.60, 0.86)  0.84 (0.73, 0.91)  0.64 (0.40, 0.79)  0.76 (0.60, 0.87)  0.69 (0.49, 0.83)  0.74 (0.57, 0.85)  0.78 (0.63, 0.87)  0.49 (0.15, 0.71)  0.69 (0.48, 0.82)  0.83 (0.71, 0.90)  0.67 (0.45, 0.81) |
| MRH | *PC scores of Joint angle and angular changes:*  PC1  PC2  PC3  PC4  PC5  PC6  PC7  PC8  PC9  PC10  PC11  PC12  PC13  PC14  PC15  PC16  PC17  PC18  PC19  PC20  PC21  PC22  PC23  PC24  PC25  PC26 | -0.2 ± 1.9  0.2 ± 1.6  0.1 ± 1.7  -0.1 ± 1.4  -0.3 ± 1.2  -0.0 ± 1.1  -0.0 ± 1.1  0.0 ± 1.0  -0.1 ± 0.9  0.2 ± 0.9  -0.1 ± 0.7  -0.0 ± 0.7  -0.1 ± 0.9  -0.0 ± 0.6  -0.1 ± 0.5  0.0 ± 0.4  0.0 ± 0.4  0.0 ± 0.3  0.0 ± 0.3  0.0 ± 0.3  0.0 ± 0.2  -0.0 ± 0.2  0.0 ± 0.2  -0.0 ± 0.1  0.0 ± 0.1  -0.0 ± 0.1 | 0.88 (0.80, 0.93)  0.89 (0.81, 0.94)  0.94 (0.90, 0.96)  0.88 (0.81, 0.93)  0.90 (0.83, 0.94)  0.90 (0.83, 0.94)  0.92 (0.87, 0.95)  0.89 (0.81, 0.94)  0.90 (0.83, 0.94)  0.88 (0.80, 0.93)  0.81 (0.67, 0.89)  0.88 (0.80, 0.93)  0.92 (0.87, 0.96)  0.91 (0.85, 0.95)  0.83 (0.72, 0.90)  0.81 (0.68, 0.89)  0.85 (0.75, 0.92)  0.71 (0.51, 0.83)  0.81 (0.68, 0.89)  0.79 (0.65, 0.88)  0.72 (0.53, 0.84)  0.78 (0.63, 0.87)  0.80 (0.66, 0.88)  0.79 (0.66, 0.88)  0.83 (0.71, 0.90)  0.79 (0.65, 0.88 | 0.1 ± 1.8  0.2 ± 1.7  0.3 ± 1.5  -0.1 ± 1.2  -0.2 ± 1.3  0.1 ± 1.2  0.1 ± 1.1  0.1 ± 1.1  -0.2 ± 0.9  0.0 ± 0.9  0.1 ± 0.6  0.0 ± 0.9  0.1 ± 0.8  0.0 ± 0.8  0.0 ± 0.5  -0.0 ± 0.4  -0.0 ± 0.4  -0.0 ± 0.4  -0.0 ± 0.3  0.0 ± 0.2  -0.0 ± 0.3  0.0 ± 0.2  -0.0 ± 0.2  0.0 ± 0.1  0.0 ± 0.1  0.0 ± 0.1 | 0.87 (0.78, 0.93)  0.91 (0.84, 0.95)  0.88 (0.79, 0.93)  0.89 (0.82, 0.94)  0.92 (0.87, 0.96)  0.91 (0.85, 0.95)  0.86 (0.76, 0,92)  0.89 (0.81, 0.94)  0.89 (0.82, 0.94)  0.87 (0.78, 0.92)  0.75 (0.57, 0.86)  0.83 (0.72, 0.90)  0.88 (0.80, 0.93)  0.92 (0.87, 0.96)  0.72 (0.53, 0.84)  0.72 (0.53, 0.84)  0.84 (0.73, 0.91)  0.80 (0.66, 0.88)  0.81 (0.68, 0.90)  0.68 (0.46, 0.82)  0.80 (0.67, 0.89)  0.76 (0.59, 0.86)  0.86 (0.76, 0.92)  0.75 (0.59, 0.86)  0.80 (0.67, 0.90)  0.68 (0.46, 0.82) |
| COH | *PC scores of Joint angle and angular changes:*  PC1  PC2  PC3  PC4  PC5  PC6  PC7  PC8  PC9  PC10  PC11  PC12  PC13  PC14  PC15  PC16  PC17  PC18  PC19  PC20  PC21  PC22  PC23  PC24  PC25  PC26 | -0.2 ± 1.8  0.1 ± 1.6  0.2 ± 1.6  -0.0 ± 1.7  -0.3 ± 1.3  -0.3 ± 1.5  -0.1 ± 1.4  0.1 ± 1.2  -0.1 ± 1.2  0.2 ± 1.3  -0.0 ± 1.0  -0.1 ± 1.0  0.1 ± 1.0  -0.1 ± 0.7  -0.1 ± 0.6  0.1 ± 0.6  0.1 ± 0.5  -0.1 ± 0.4  -0.0 ± 0.4  -0.0 ± 0.3  -0.1 ± 0.3  -0.0 ± 0.2  0.0 ± 0.2  -0.0 ± 0.2  0.0 ± 0.1  -0.0 ± 0.2 |  | -0.2 ± 1.7  0.3 ± 1.7  0.3 ± 1.7  -0.1 ± 1.5  -0.1 ± 1.4  0.1 ± 1.4  0.0 ± 1.3  0.1 ± 1.0  -0.0 ± 1.2  -0.0 ± 0.9  -0.1 ± 0.8  -0.0 ± 1.0  -0.1 ± 0.9  -0.0 ± 0.7  0.1 ±0.6  -0.1 ± 0.5  0.0 ± 0.5  -0.0 ± 0.4  -0.0 ± 0.5  -0.1 ± 0.4  -0.0 ± 0.3  -0.0 ± 0.3  -0.0 ± 0.2  0.0 ± 0.1  0.0 ± 0.1  -0.0 ± 0.1 |  |

SLH = single leg hop; CMJ = countermovement jump; MRH = 90° medial rotational hop; COH = cross-over hop
